# Protective effects of solvent fractions of *Mentha spicata* (L.) leaves evaluated on 4-nitroquinoline-1-oxide induced chromosome damage and apoptosis in mouse bone marrow cells

**DOI:** 10.1590/S1415-47572009005000086

**Published:** 2009-12-01

**Authors:** Ponnan Arumugam, Arabandi Ramesh

**Affiliations:** 1 Department of Genetics, Dr. A.L.M. Post Graduate Institute of Basic Medical Sciences, University of MadrasIndia; 2 Department of Environmental and Occupational Health, National Cheng Kung University Medical College, National Cheng Kung University, TainanTaiwan

**Keywords:** 4-NQO, chromosome damage, *Menthaspicata* protective effects

## Abstract

Spearmint leaves (*Mentha spicata* L.) contain high levels of antioxidants that are known to protect against both exogenous and endogenous DNA damage. In this study, the protective effects of the hexane fraction (HF), chloroform fraction (CF) and ethyl acetate fraction (EAF) in an ethanol extract from *M. spicata* were evaluated against 4-nitroquinoline-1-oxide (4-NQO) induced chromosome damage and apoptosis in bone marrow cells of Swiss albino mice. Two (EAF; 80 and 160 mg/ kg body weight - bw) or three (HF and CF; 80, 160 and 320 mg/ kg bw) doses of solvent fractions or vehicle control (25% DMSO in water) were administered orally for five consecutive days. Upon the sixth day, 4-NQO was injected intraperitoneally. The animals were killed the following day. Other control groups were comprised of animals treated with either the vehicle control or the various doses of solvent fractions, but with no 4-NQO treatment. 4-NQO induced micro-nucleated polychromatic erythrocytes (MnPCEs) in all the test groups. However, pre-treatment of animals with the solvent fractions significantly reduced the 4-NQO-induced MnPCEs as well as the percentage of apoptotic cells. The reduction of both MnPCE and apoptosis was more evident following the pre-treatment of animals with 160 mg/kg bw EAF.

## Introduction

*Mentha spicata* L. (Lamiaceae) is commonly known as spearmint and in India as “*Pudina*” (Ali *et al.*, 2006). The leaves are well recognized for making a herbal tea and for use in the preparation of folk medicine (Carmona *et al.*, 2005; Adsersen *et al.*, 2006). Derived spearmint oils are used for the manufacture of food confectioneries and pharmaceuticals (Tognolini *et al.*, 2006). The plant is known to be endowed with a variety of biological properties due to the high content of secondary metabolites (Choudhury *et al.*, 2006). It was found to be anti-allergic (Yamamura *et al.*, 1998), anti-oxidant (Kanatt *et al.*, 2007), anti-platelet (Tognolini *et al.*, 2006), anti- cytotoxic (Manosroi *et al.*, 2006) and chemo-preventive (Saleem *et al.*, 2000), besides exerting H_2_O_2_ scavenging activities (Kumar and Chattopadhyay, 2007). It also possesses *in vitro* anti-mutagenic activity against the direct acting mutagen, 2-hydroxy amino-3-ethyl-3*H*-imidazo [4, 5-*f*] quinoline (*N*-OH-IQ) (Yu *et al.*, 2004). Recently, we found that *M. spicata* contains a potent antioxidant and excercises anti-inflammatory activities (Arumugam *et al.*, 2006, 2008a).

When subjected to the Ames, chromosomal aberration and micronucleus tests, 4-nitroquinoline-1-oxide (4-NQO) is revealed as a well-known carcinogen and mutagen. 4-NQO exerts mutagenesis through its metabolite 4-hydroxyaminoquinoline-1-oxide (4HAQO). 4-NQO is converted into 4-HAQO and 4- aminoquinoline-1-oxide (4AQO) by DT-diaporases in the presence of NADPH (Papp-Szabo *et al.*, 2003). 4-HAQO intercalates with DNA through the covalent bond and can form three types of adducts, two on guanine (dGuo-N2-AQO, dGuo-C8-AQO) and one on adenine (dAdo-N6-AQO). 4-NQO also forms pyrimidine dimmers and produces bulky DNA adducts, as is the case of UV light does (Kanojia and Vaidya, 2006). Moreover, 4-NQO causes oxidative DNA damage by producing intracellular oxidative stress through the generation of reactive oxygen species (ROS), superoxides and hydroxyl radicals when undergoing the redox-cycle. A high level of oxidative stress (ROS) induces cytotoxicity, DNA strand breaks and mutations (Papp-Szabo *et al.*, 2003).

In the present study, the protective effects of various solvent fractions of *Mentha spicata* on 4-NQO induced chromosome damage and apoptosis in mouse bone-marrow cells were investigated.

## Materials and Methods

###  Animals

Swiss albino mice of either sex, 10 to 12 weeks old and 25-30 grams were used. All animals were obtained from the King Institute, Chennai, India, and were kept in the Institute's animal house under standard environmental conditions (temperature: 22 ± 2 °C and 12 h light/ dark period). Their maintenance was in accordance with the guidelines of the Committee for the Purpose of Control and Supervision of Experiments on Animals (CPCSEA), Government of India. The Institute's ethical committee approved all the experiments.

###  Chemicals

4-Nitroquinoline N-oxide (4-NQO), Giemsa stain, May-Grunwald stain and Annexin V-FITC assay kit were purchased from Sigma-Aldrich, USA. All the other solvents used in the experiments were of analytical grade.

###  Procedure for solvent fractionation

*M. spicata* L. was commercially purchased and identified at the Center for Advanced Studies in Botany, University of Madras (voucher number-855). The different solvent fractions (SF) such as the hexane fraction (HF), chloroform fraction (CF) and ethyl acetate fraction (EAF) were fractionated from the ethanol extract of dried-leaf powder of *M. spicata* by the Villasenor *et al.* (2003) method. The procedure was briefly given in our previous paper (Arumugam *et al.*, 2008a).

###  Experimental design for the micronucleus test

The test doses of solvent fractions such as HF, CF and EAF were determined on the basis of their estimated LD_50_ doses and on preliminary laboratory experiments. These doses corresponded to 50, 25 and 12.5% of the LD_50_ (Table 1).

The femurs from each animal were dissected and the proximal heads removed. The contents of both femurs were flushed out and pooled into a total volume of 3 mL pre-filtered foetal-calf serum. The cell suspensions were sedimented by centrifugation, the supernatant discarded and the cells themselves resuspended in a small volume of fresh foetal calf serum. A droplet from the suspension was transferred to a glass microscope slide and a smear prepared. At least four smears were prepared from each animal and scored according to standard May-Grunwald staining (Schmid, 1975).

###  Experimental design for detection of apoptotic cells

The maximum effective doses of solvent fractions against 4-NQO induced MnPCEs were used. The experiments consisted of four groups, each group being comprised of six mice of either sex (Table 2).

**Figure 1:**
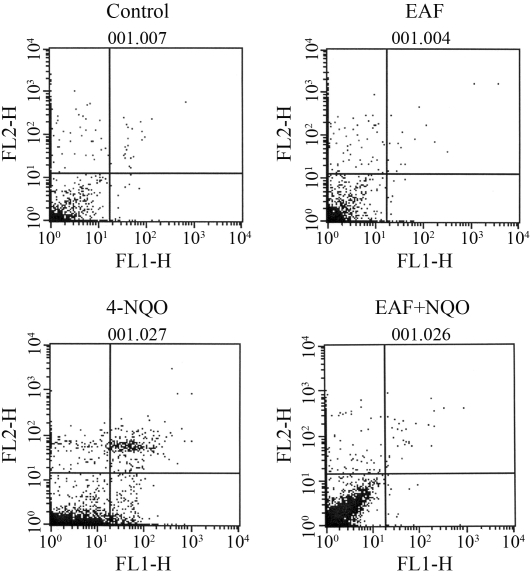
Effects of the ethyl acetate fraction (EAF) on cytometric flow in mouse bone-marrow cells with annexin V-FITC (FL1-H) *vs.* PI (FL2-H). Lower left: non-apoptotic live cells (annexin V-FITC^-ve^ and PI^-ve^). Lower right: early apoptotic cells (annexin V-FITC^+ve^ and PI^-ve^). Upper right: Late apoptotic and necrotic cells (annexin V-FITC^+ve^ and PI^+ve^). Upper left: damaged cells (annexin V- FITC^-ve^ and PI^+ve^).

Both femurs were removed and the bone marrow cells flushed out with 2 mL of phosphate buffered saline (PBS). These cells were then washed twice with PBS by gentle aspiration. The pellet was obtained following centrifugation for 5 min at 2000 rpm (4 °C), and was then re-suspended with 500 μL of fresh PBS. According to annexin V- FITC assay kit procedure, 1 x 10^6^ cells were placed into one mL of binding buffer, to which 5 μL of annexin V - FITC were added, followed by 10 μL of propidium iodide (PI- 20 μg per mL). After mixing the cell suspension, the cells were incubated in the dark for 15 min at room temperature. They were immediately analyzed by flow cytometry (Darzynkiewicz *et al.*, 2001). Four different groups of cells were measured (Figure 1). The total number of apoptotic cells was calculated by the addition of both early and late apoptotic ones.

###  Statistical analysis

Results were presented as the mean ± standard error for six mice of each group. Statistical analyses were performed by one-way ANOVA using SPSS Software Version 12.0. The Student-Neuman-Keuls test (SNK TEST) was applied to assess for differences among the groups. Values of p ≤ 0.05 were considered to be significant.

## Results

The protective effects of solvent fractions of *M. spicata* against 4-NQO induced micro-nucleated polychromatic erythrocytes (MnPCEs) of mouse bone-marrow are presented in Table 3. The genotoxin 4-NQO enhanced MnPCE frequency by ~4 times the control value, 15.78 MnPCEs/2500 PCEs. Treatment with solvent fractions alone was ineffectual against MnPCE frequency (p > 0.05). Pre-treatment with the hexane fraction significantly reduced the mutation rate from about 58 to 42%. Dose differences were significant only at higher doses. The chloroform fraction also significantly reduced 4-NQO induced mutation frequency. The reduction was greatest at 320 mg per kg bw (about 61%) and lowest at 80 mg per kg bw (about 45%). Nevertheless, the ethyl acetate fraction showed the highest reduction of MnPCEs induced by 4-NQO at half the dose, *i.e.* 160 mg per kg bw when compared to the other two fractions. Even the lowest dose, 80 mg per kg bw was also effective in reducing MnPCE frequency (about 51%) which was comparable to the CF and higher than the HF at their dose of 160 mg per kg bw.

**Figure 2:**
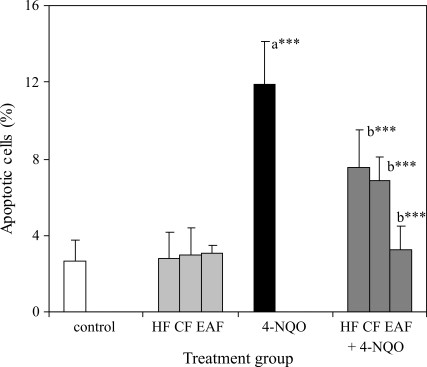
Protective effects of solvent fractions of *M. spicata* against 4-NQO-triggered apoptosis in mouse bone-marrow cells (4-NQO: 4-nitroquinoline-1-oxide) HF - hexane fraction, CF - chloroform fraction, EAF - ethyl acetate fraction. Data were expressed as mean ± standard error (*n* = 6); Significant difference at p < 0.001^***^ and p < 0.05^*^ (Student-Newman-Keuls), ‘a' stands for comparison with the control group, ‘b' stands for comparison with the only-4-NQO group).

Moreover, the study of flow cytometry gave support to the proposition that 4-NQO induced chromosome damage in bone-marrow cells. Four groups of cells were estimated by using the annexin V-FITC assay kit (Figure 1). The total of apoptotic cells, including those of both early and late apoptosis, was arrived at after 24 h of treatment with 4-NQO (Figure 2). The total of apoptotic cells was found to be 12% superior to that of the control (2.65%). Treatment with solvent fractions alone did not reveal any effect on bone-marrow apoptosis. However, pretreatment with solvent fractions showed statistically significant decreases in apoptotic cells induced by 4-NQO. The highest reduction was also found to be in the EAF (3.24%) followed by CF (6.87%) and HF (7.51%). Overall, the results indicated that EAF was more effective against 4-NQO induced chromosome damage than the other two fractions.

## Discussion

The protective effect of solvent fractions of *M. spicata* was evaluated against 4-nitroquinoline-1-oxide (4-NQO) induced chromosome damage in mice bone marrow. 4-NQO is a potent mutagen that exerts its genotoxicity in two ways. In the first and during metabolism, 4-NQO is converted into 4-hydroxyaminoquinoline 1-oxide (Ac-4-HAQO) and interacts with DNA at N2 and C8 of guanosine and also at N6 of adenine forming adducts (Han *et al.*, 2007). As a consequence, the helical structure of DNA changes, resulting in micronuclei/chromosomal breakage (Diekmann *et al.*, 2004). The second pathway occurs through oxidative stress caused by the formation of reactive oxygen species (ROS), such as superoxide and hydroxyl radicals, whereat 4-NQO undergoes redox cycling that brings about modified bases and DNA strand breaks (Zhang *et al.*, 2008). Hence, 4-NQO enhanced the frequency of MnPCEs by about four times that of the control group (15.78 MnPCEs/2500 PCEs; Table 3). The observed PCE/NCE ratio is presented in Table 4. The 4-NQO group is the only one with a significant reduction in PCE/NCE ratio compared to the control. Pre-treatment with solvent fractions effectively decreased 4-NQO enhanced MnPCE frequency by about 42%-65%, depending on the dose and solvent fraction tested. Moreover, the reduction of MnPCE by solvent fractions corresponded well with their PCE/NCE ratio. The maximum reduction of 58% was observed for HF and 61% for CF at the dose of 320 mg per kg bw. Thus, there were large amounts of pigments and less phenol in the two fractions (Arumugam *et al.*, 2008b). Pigments (carotenoids and xanthophylls) are known to exert antimutagenic and anticarcinogenic activities either by trapping 4-NQO intermediates or by the inhibition/degradation of its metabolizing enzymes. Ferraz *et al.*, (2005) observed that lower polar fractions, such as hexane and chloroform, exerted more effective anti-proliferation due to pigments than higher polar fractions (ethyl acetate).

Among the solvent fractions, the highest reduction of MnPCEs (about 65%), was observed for EAF at a dose of 160 mg per kg bw. Even at a lower dose (80 mg per kg bw), EAF possesses a more pronounced antigenotoxic effect when compared to the highest doses of CF and HF. In contrast, CF from the ethanol extract of *M*. *cordifolia* was shown to exercise the highest MnPCE reduction against tetracycline-induced genotoxicity followed by EAF and HF. The efficacy of CF was reported to be in the presence of β-sitosterol (Villasenor *et al.*, 2003). Hence, we found that as to the protective effect of solvent fractions against 4-NQO induced chromosome damage, the order was EAF > CF > HF, which was well in accordance with their corresponding order of antioxidants and secondary metabolites. The effectiveness of EAF could be due to the large phenol and flavonoid content (Arumugam *et al.*, 2008b). They are all well known antioxidants involved in protecting against DNA damage induced by ROS (Kumar and Chattopadhyay, 2007). In a previous study, it was also observed that EAF displayed more intense anti-inflammatory activity than the other fractions (Arumugam *et al.*, 2008a).

Furthermore, data from the study on flow cytometry also gave support to the concept on the protective effects of solvent fractions on 4-NQO induced chromosome damage. DNA damage is known to be one of the hallmarks for the formation of apoptosis (Mazur *et al.*, 2002). During genotoxic stress, cells undergo apoptosis in the case of DNA damage being beyond repair. 4-NQO can induce apoptosis by way of the p53-dependent mitochondrial signaling pathway (Han *et al.*, 2007). The results clearly indicated that MnPCEs induced by 4-NQO (~4 times higher than control - Table 3) was reflected in the form of total apoptotic cells (~4 times higher than control value; Figure 2). Even, the PCE/NCE ratio was also reflected by 4-NQO induced apoptotic cells (Table 4). However, pre-treatment with EAF significantly reduced 4-NQO induced apoptosis to a greater degree than with the other two fractions.

In summary, among the three solvent fractions, EAF exerted the highest protective effect against 4-NQO induced chromosome damage in mice, since EAF was found to be a powerful antioxidant due to the high level of polyphenols (Arumugam *et al.*, 2006), this maybe constituting the means of protecting bone-marrow cells in mice from 4-NQO toxicity. On other hand, polyphenols have been reported to inhibit 4-NQO induced free radicals (Srinivasan *et al.*, 2007). Hence, further study is called for in order to clarify what is the mechanism of EAF against 4-NQO induced chromosome damage in mice.

## Figures and Tables

**Table 1:** Experimental design for micronucleus test. Solvent fractions were administered orally to mice; 4-NQO treatment was performed on day 6 by intraperitoneal injection, and animals were killed on day 7.

Treatment group	Treatment on days 1 to 5	Number of mice	
		Males	Females
Vehicle	25% DMSO	3	3
HF	80 mg per kg bw	3	3
HF	160 mg per kg bw	3	3
HF	320 mg per kg bw	3	3
CF	80 mg per kg bw	3	3
CF	160 mg per kg bw	3	3
CF	320 mg per kg bw	3	3
EAF	80 mg per kg bw	3	3
EAF	160 mg per kg bw	3	3
4-NQO	7.5 mg per kg bw	3	3
HF +4-NQO	80 +7.5 mg per kg bw	3	3
HF+4-NQO	160 +7.5 mg per kg bw	3	3
HF+4-NQO	320 +7.5 mg per kg bw	3	3
CF+4-NQO	80+7.5 mg per kg bw	3	3
CF+4-NQO	160 +7.5 mg per kg bw	3	3
CF+4-NQO	320+7.5 mg per kg bw	3	3
EAF+4-NQO	80 +7.5 mg per kg bw	3	3
EAF+4-NQO	160 +7.5 mg per kg bw	3	3

HF, hexane fraction, CF chloroform fraction, and EAF ethyl acetate fraction of ethanol extract from *M. spicata*; 4-NQO, 4-nitroquinoline-1-oxide.

**Table 2:** Experimental design for the detection of apoptotic cells. The solvent fractions were fed orally to mice, 4-NQO treatment was performed on day 6 by intraperitoneal injection, and the animals were killed on day 7.

Treatment group	Treatment on days 1 to 5	Number of mice	
		Males	Females
Vehicle	25% DMSO	3	3
HF	320 mg per kg bw	3	3
CF	320 mg per kg bw	3	3
EAF	160 mg per kg bw	3	3
4-NQO	7.5 mg per kg bw	3	3
HF+4-NQO	320 +7.5 mg per kg bw	3	3
CF+4-NQO	320+7.5 mg per kg bw	3	3
EAF+4-NQO	160 +7.5 mg per kg bw	3	3

HF, hexane fraction, CF chloroform fraction, and EAF ethyl acetate fraction of ethanol extract from *M. spicata*; 4-NQO, 4-nitroquinoline-1-oxide.

**Table 3:** Modulation of 4-NQO induced chromosome damage in mice pre-treated with solvent fractions from *M. spicata.*

Treatment groups (dose: mg per kg bw)	HF			CF			EAF	
	(MnPCEs/ 2500 PCEs)	Decrease (%)		(MnPCEs/ 2500 PCEs)	Decrease (%)		(MnPCEs/ 2500 PCEs)	Decrease (%)
Control	15.78 ± 0.87	-		15.78 ± 0.87	-		15.78 ± 0.87	-
80	14.60 ± 0.86	-		17.97 ± 2.77	-		16.63 ± 1.26	-
160	10.03 ± 1.11	-		15.85 ± 2.60	-		14.70 ± 1.14	-
320	9.07 ± 1.35	-		13.82 ± 2.48	-		-	-
4-NQO (7.5)	67.12 ± 4.93^a***^	100.0		67.12 ± 4.93^a***^	100.00		67.12 ± 4.93^a***^	100.0
80 + 4-NQO (7.5)	38.82 ± 1.60^b^***	42.16		36.73 ± 3.27^b^***	45.28		32.98 ± 3.39^b^***	50.86
160 + 4-NQO (7.5)	35.43 ± 1.94^b^***	47.21		31.97 ± 3.61^b^***	52.37		23.38 ± 2.19^b^***	65.17
320 + 4-NQO (7.5)	28.10 ± 1.55^b^***	58.14		26.05 ± 1.91^b^***	61.19		-	-

4-NQO - 4-nitroquinoline-1-oxide; HF - hexane fraction; CF - chloroform fraction; EAF - ethyl acetate fraction; MnPCEs - micronucleated polychromatic erythrocytes, expressed: Mean ± standard error (*n* = 6), significant difference at p < 0.001^***^ and p < 0.05^*^ (Student-Newman-Keuls); ‘a' stands for comparison with the control group, ‘b' stands for comparison with the only-4-NQO group; decrease (%): over the 4-NQO induced MnPCEs.

**Table 4:** Ratio of PCE/NCE induced by 4-NQO in mice pre-treated with solvent fractions from *M. spicata.*

Treatment groups (dose: mg per kg bw)	HF PCE/NCE	CF PCE/NCE	EAF PCE/NCE
Control	1.30	1.41	1.41
80	1.25	1.50	1.58
160	1.39	1.67	1.38
320	1.52	1.75	-
4-NQO (7.5)	0.75	0.75	0.75
80 + 4-NQO (7.5)	0.92	0.90	0.84
160 + 4-NQO (7.5)	0.91	0.95	1.02
320 + 4-NQO (7.5)	1.04	0.89	-

PCE: Polychromatic erythrocytes; NCE: normochromatic erythrocytes; 4-NQO: 4-nitroquinoline-1-oxide; HF: hexane fraction; CF: chloroform fraction; EAF: ethyl acetate fraction.

## References

[Adsersenetal2006] Adsersen A., Gauguin B., Gudiksen L., Jäger A.K. (2006). Screening of plants used in Danish folk medicine to treat memory dysfunction for acetylcholinesterase inhibitory activity. J Ethnopharmacol.

[Alietal2006] Ali M.S., Ahmed W., Saleem M., Khan T. (2006). Longifoamide-A and B: Two new ceramides from *Mentha longifolia* (Lamiaceae). Nat Prod Res.

[Arumugametal2006] Arumugam P., Ramamurthy P., Santhiya S.T., Ramesh A. (2006). Antioxidant activity measured in different solvent fractions obtained from *Mentha spicata* Linn. : Ananalysis by ABTS.+ decolorization assay. Asia Pacif J Clin Nutr.

[Arumugametal2008a] Arumugam P., Gayatri Priya N., Subathra M., Ramesh A. (2008a). Anti-inflammatory activity of four solvent fractions of ethanol extract of *Mentha spicata* L. investigated on acute and chronic inflammation induced rats. Environ Toxicol Pharmacol.

[Arumugametal2008b] Arumugam P., Ramamurthy P., Ramesh A. (2008b). Antioxidant and cytotoxic activities of lipophilic and hydrophilic fractions of *Mentha spicata* L. (Lamiaceae). Int J Food Property.

[Carmonaetal2005] Carmona M.D., Llorach R., Obon C., Rivera D. (2005). “Zahraa”, a Unani multicomponent herbal tea widely consumed in Syria: Components of drug mixtures and alleged medicinal properties. J Ethnopharmacol.

[Choudhuryetal2006] Choudhury R.P., Kumar A., Garg A.N. (2006). Analysis of Indian mint (*Mentha spicata*) of essential, trace and toxic elements and its antioxidant behaviour. J Pharmaceut Biomed Anal.

[Darzynkiewiczetal2001] Darzynkiewicz Z., Bedner E., Smolewski P. (2001). Flow cytometry in analysis of cell cycle and apoptosis. Sem Hematol.

[Diekmannetal2004] Diekmann M., Waldmann P., Schnurstein A., Grummtd T., Braunbeck T., Nagel R. (2004). On the relevance of genotoxicity for fish populations II: Genotoxic effects in zebrafish (*Danio rerio*) exposed to 4-nitroquinoline-1-oxide in a complete life-cycle test. Aquat Toxicol.

[Ferrazetal2005] Ferraz A., Faria D.H., Benneti M.N., Rocha A.B., Schwartsmann G., Henriques A., Poser G.L. (2005). Screening for antiproliferative activity of six southern Brazilian species of *Hypericum*. Phytomedicine.

[Hanetal2007] Han H., Pan Q., Zhang B., Li J., Deng Z., Lian Z., Li N. (2007). 4-NQO induces apoptosis via p53-dependent mitochondrial signaling pathway. Toxicology.

[Kanattetal2007] Kanatt S.R., Chander R., Sharma A. (2007). Antioxidant potential of mint (*Mentha spicata* L. ) in radiation-processed lamb meat. Food Chem.

[KanojiaandVaidya2006] Kanojia D., Vaidya M.M. (2006). 4-Nitroquinoline-1-oxide induced experimental oral carcinogenesis. Oral Oncol.

[KumarandChattopadhyay2007] Kumar A., Chattopadhyay S. (2007). DNA damage protecting activity and antioxidant potential of pudina extract. Food Chem.

[Manosroietal2006] Manosroi J., Dhumtanom P., Manosroi A. (2006). Anti-proliferative activity of essential oil extracted from Thai medicinal plants on KB and P388 cell lines. Cancer Lett.

[Mazuretal2002] Mazur L., Augustynek A., Bochenek M. (2002). Flow cytometric estimation of the plasma membrane diversity of bone marrow cells in mice treated with WR-2721 and cyclophosphamide. Toxicology.

[Papp-Szaboetal2003] Papp-Szabo E., Douglas G.R., Coomber B.L., Josephy P.D. (2003). Mutagenicity of the oral carcinogen 4-nitroquinoline-1-oxide in cultured BigBlue rat tongue epithelial cells and fibroblasts. Mutat Res.

[Saleemetal2000] Saleem M., Alam A., Sultana S. (2000). Attenuation of benzoyl peroxide-mediated cutaneous oxidative stress and hyperproliferative response by the prophylactic treatment of mice with spearmint (*Mentha spicata*). Food Chem Toxicol.

[Schmid1975] Schmid W. (1975). The micronucleus tests. Mutat Res.

[Srinivasanetal2007] Srinivasan P., Sabitha K.E., Shyamaladevi C.S. (2007). Attenuation of 4-Nitroquinoline 1-oxide induced *in vitro* lipid peroxidation by green tea polyphenols. Life Sci.

[Tognolinietal2006] Tognolini M., Barocelli E., Ballabeni V., Bruni R., Bianchi A., Chiavarini M., Impicciatore M. (2006). Comparative screening of plant essential oils: Phenylpropanoid moiety as basic core for antiplatelet activity. Life Sci.

[Villasenoretal2003] Villasenor I.M., Echegoyen D.E., Angeladia J.S. (2003). A new antimutagen from *Mentha cordifolia* Opiz. Mutat Res.

[Yamamuraetal1998] Yamamura S., Ozawa K., Ohatani K., Kasai R., Yamasaki K. (1998). Antihistaminic flavones and aliphatic glycosides from *Mentha**spicata*. Phytochemistry.

[Yuetal2004] Yu T., Xu M., Dashwood R.H. (2004). Antimutagenic activity of Spearmint. Environ Mol Mutagen.

[Zhangetal2008] Zhang H., Wang J., Li R., Bai J., Ye Y., Ren F. (2008). Attenuated effects of peptides derived from porcine plasma albumin on *in vitro* lipid peroxidation in the liver homogenate of mice. Food Chem.

